# Kaempferol Attenuates Oxidative Stress‐Induced Injury in Gastric Mucosal Cells by Activating Nrf2/GPX4 Axis to Inhibit Ferroptosis

**DOI:** 10.1002/iid3.70352

**Published:** 2026-02-12

**Authors:** Chao Luo, Jing Yan, Yun Shen, Zhiguang Sun, Xiong Xiao

**Affiliations:** ^1^ Department of Gastroenterology The Second Affiliated Hospital of Nanjing University of Chinese Medicine (The Second Hospital of Jiangsu Province of TCM) Nanjing Jiangsu People's Republic of China; ^2^ Medical Research Center of First College of Clinical Medicine Nanjing University of Chinese Medicine Nanjing Jiangsu People's Republic of China; ^3^ First Clinical Medical College Nanjing University of Chinese Medicine Nanjing Jiangsu People's Republic of China

**Keywords:** ferroptosis, gastric mucosal injury, Kaempferol, Nrf2/GPX4, oxidative stress

## Abstract

**Objective:**

This study explores how Kaempferol (KAE) protects against oxidative stress‐induced damage by suppressing ferroptosis via the Nrf2/GPX4 axis in gastric mucosal cells.

**Methods:**

Human gastric epithelial cells (GES‐1) were treated with H₂O₂ to induce oxidative damage, following pretreatment with varying doses of KAE. Cell vitality was assessed by the CCK‐8 experiment, apoptosis was monitored using flow cytometry, and intracellular ROS levels and lipid peroxidation were determined by fluorescence probes. Intracellular malondialdehyde (MDA), glutathione (GSH/GSSG ratio), and Fe²⁺ were measured using biochemical assays. Expression and cellular distribution of Nrf2, GPX4, SLC7A11, and ACSL4 were assessed using Western blot analysis and immunofluorescence techniques. Additionally, the Nrf2‐specific inhibitor ML385 was employed to confirm the role of the Nrf2/GPX4 axis.

**Results:**

KAE (0–40 μM) was non‐toxic and enhanced GES‐1 cell viability under H₂O₂‐induced stress, with optimal protection at 10 μM. It reduced ROS, lipid peroxidation, MDA, and Fe²⁺ levels, while increasing the GSH/GSSG ratio. KAE also influenced ferroptosis‐associated proteins by increasing GPX4 and SLC7A11 expression while reducing ACSL4 levels. Additionally, it promoted Nrf2 nuclear translocation. These effects were attenuated by the Nrf2 inhibitor ML385, indicating involvement of the Nrf2/GPX4 axis.

**Conclusion:**

KAE protects against H₂O₂‐induced gastric epithelial damage through activating the Nrf2/GPX4 axis, thereby lowering oxidative injury and ferroptotic processes, and offering a potential therapeutic strategy for gastric mucosal protection.

## 1 Introduction

Gastric ulcers are frequently occurring gastrointestinal disorders resulting from damage to the gastric mucosal barrier, which allows gastric acid and enzymes to erode the stomach wall. Normally, mucus and bicarbonate secretion protect the mucosa, but factors such as *H. pylori* infection, intake of nonsteroidal anti‐inflammatory drugs, and unhealthy diet—can disrupt this defense, resulting in gastritis or peptic ulcer [1]. Epidemiological data indicate that the global lifetime prevalence of peptic ulcer disease is about 5%–10%, with an annual incidence of 0.3%−1.9% [2, 3]. Gastric mucosal injury can result in serious complications such as bleeding and perforation. Despite advances in therapy, the related complications remain severe, the incidence of perforation can reach 50%, with a mortality rate up to 30% [4], potentially endangering patient health.

Oxidative stress is critically involved in gastric mucosal injury progression. Overproduction of reactive oxygen species (ROS) triggers lipid peroxidation, DNA damage, and apoptosis, compromising mucosal integrity [5]. Ferroptosis, an iron‐ and lipid peroxide‐dependent type of programmed cell death [6], has recently attracted attention. Studies indicate that oxidative stress can promote lipid peroxidation and iron accumulation, thereby triggering ferroptosis and worsening mucosal injury [7, 8]. The interaction of oxidative stress and ferroptosis results in membrane rupture and cell death, further impairing gastric mucosal integrity [9]. Thus, targeting these pathways may offer potential strategies for managing gastric mucosal damage.

Nuclear factor erythroid 2‐related factor 2 (Nrf2) is central to cellular antioxidant defense. In response to oxidative stress, Nrf2 disengages from Keap1, migrates into the nucleus, and stimulates transcription of antioxidant defense genes [10]. Glutathione peroxidase 4 (GPX4), a key downstream target of Nrf2, eliminates lipid peroxides and prevents ferroptosis [11]. Evidence shows that Nrf2 regulates GPX4 expression protects against ferroptosis in various diseases [12, 13], highlighting the importance of the Nrf2/GPX4 axis in cell protection.

Kaempferol (KAE), a natural flavonoid found in several medicinal plants such as *Cyperus rotundus* and *Paeonia lactiflora*, exhibits antioxidative and anti‐inflammatory properties [14]. Recent studies demonstrate that it is not only beneficial in atherosclerosis [15], ischemia‐reperfusion injury [16], but also regulates gut microbiota, enhances intestinal barrier function, and alleviates intestinal inflammation [17, 18], which underscores its potential in the prevention and treatment of gastric mucosal injury and related gastrointestinal diseases. Notably, KAE can inhibit Fe²⁺‐induced lipid peroxidation and scavenge free radicals [19]. Additionally, emerging evidence suggests that KAE modulates Nrf2‐related pathways to alleviate oxidative damage in various disease models [20, 21]. However, to date, no studies have documented the protective role of KAE in gastric mucosal injury through modulation of the Nrf2/GPX4 axis.

Overall, given the interplay between oxidative stress, ferroptosis, and gastric mucosal damage, along with the regulatory function of the Nrf2/GPX4 axis in ferroptosis, KAE is likely to alleviate such injury through Nrf2/GPX4 activation and ferroptosis inhibition. This study seeks to clarify the mechanism by which KAE protects the gastric mucosa, offering both theoretical insight and experimental support for the development of novel mucosal protectants.

## 2 Materials and Methods

### 2.1 Cell Culture and Treatment

Gastric Epithelial Cells (GES‐1) cells (iCell Bioscience, China) were used at passages 3–10 and maintained in RPMI‐1640 medium (Sangon, China) with 10% fetal bovine serum and 1% penicillin−streptomycin, under standard conditions (37°C, 5% CO₂, humidified incubator). For experiments, cells were seeded at 5 × 10³ cells/well in 96‐well plates for viability assays or 2 × 10⁵ cells/well in six‐well plates for other analyses, and allowed to adhere overnight before treatment.

To establish an oxidative stress injury model, a 6 h exposure to 100 μM H₂O₂ (Yuanye, China) was applied to GES‐1 cells [10]. Prior to H₂O₂ stimulation, cells were pretreated with various concentrations of KAE (0, 5, 10, 20, 40 μM; MCE, USA) for 2 h. In certain groups, the ferroptosis inhibitor Ferrostatin‐1 (Fer‐1, 1 μM, MCE) or the Nrf2 inhibitor ML385 (5 μM, MCE) was co‐incubated to verify the underlying mechanisms.

### 2.2 Cell Viability Assay

Cells were incubated with 10 μL of CCK‐8 reagent (Biosharp, China) per well for 2 h at 37°C following treatment. Absorbance was subsequently recorded at 450 nm with a microplate reader (Agilent BioTek Epoch 2, US).

### 2.3 Flow Cytometry

An Annexin V‐FITC/PI kit (KeyGEN, China) was used to detect apoptotic and necrotic cells. Briefly, cells were harvested and rinsed with pre‐chilled PBS, and the density was adjusted to 1 × 10⁶ cells/mL. Next, 5 μL of Annexin V‐FITC and PI were mixed with 100 μL of cell suspension, and incubated in darkness for 15 min. Afterwards, 400 μL of binding buffer was added, and samples were subjected to a flow cytometer (Beckman CytoFLEX, USA).

### 2.4 ROS Detection

Cells were treated with 10 μM DCFH‐DA (Uelandy, China) at 37°C for 30 min to detect intracellular ROS levels. After washing with PBS, fluorescence was observed under an Olympus BX53 fluorescence microscope (Japan). Fluorescence intensity was quantified using ImageJ software.

### 2.5 Lipid Peroxidation Assay

Lipid peroxidation was assessed using the BODIPY‐C11 probe (Thermo Fisher Scientific, USA). Cells were washed and then stained with 2 μM BODIPY‐C11 for 30 min, followed by fluorescence microscopy imaging and quantification of fluorescence intensity using ImageJ.

### 2.6 Malondialdehyde (MDA) Measurement

A lipid peroxidation assay kit (Elabscience, China) was employed to quantify MDA levels as per the guidelines. The optical density at 532 nm was recorded using a microplate reader.

### 2.7 GSH/GSSG Ratio Determination

After treatment, cells were lysed with 5% trichloroacetic acid (TCA), and the supernatant was collected via centrifugation. A commercial colorimetric kit (Elabscience, China) was employed to determine the concentrations of total glutathione (T‐GSH) and oxidized glutathione (GSSG). T‐GSH was measured based on the reaction of GSH with DTNB, producing a yellow product (TNB) with absorbance at 412 nm. GSSG was specifically measured after masking GSH with 2‐vinylpyridine (2‐VP), followed by reduction with glutathione reductase (GR) and NADPH. The concentration of reduced GSH was derived using the equation: GSH = T‐GSH − 2 × GSSG, and the GSH to GSSG ratio was subsequently calculated.

### 2.8 Intracellular Fe²⁺ Quantification

A ferrozine‐based colorimetric assay kit (Applygen, China) was used to determine free Fe²⁺ concentrations. After ultrasonic lysis, the cell supernatant was mixed with the iron detection reagent, and the absorbance was recorded at 562 nm.

### 2.9 Western Blot Analysis

Cellular proteins were isolated using RIPA buffer (Beyotime, China) containing protease inhibitors.

Nuclear and cytoplasmic proteins were separated using the Nuclear and Cytoplasmic Extraction Reagents kit (Thermo Scientific, USA) according to the manufacturer's protocol. Briefly, cells were resuspended in ice‐cold cytoplasmic extraction buffer with protease inhibitors, incubated on ice for 10 min, and centrifuged at 500 × *g* for 5 min at 4°C to collect the cytoplasmic fraction (supernatant). The pellet was then resuspended in nuclear extraction buffer, vortexed intermittently for 40 min, and centrifuged at 14,000 × *g* for 10 min at 4°C to obtain the nuclear fraction. Protein levels were determined using a BCA kit (Beyotime, China). Protein samples were resolved using SDS‐PAGE, transferred to PVDF membranes (0.45 μm, Millipore, USA), and incubated overnight at 4°C with primary antibodies (Nrf2, GPX4, SLC7A11, ACSL4, GAPDH, Histone H3). After 1 h incubation with HRP‐conjugated secondary antibodies, protein bands were detected using ECL reagents (Epizyme, China), and band intensities were quantified with ImageJ software. The intensity of each target protein band was quantified using ImageJ and normalized to the corresponding loading control (GAPDH for cytoplasmic proteins, Histone H3 for nuclear proteins) to account for loading differences. Detailed information of antibodies was listed in Table 1.

**Table 1:** Information of antibodies used in Western blot analysis and immunofluorescence staining.

Target protein	Host	Dilution	Catalog no.	Manufacturer's
Nrf2	Rabbit	1:1000 WB 1:200 IF	#12721	CST
GPX4	Rabbit	1:1000 WB 1:200 IF	ab125066	Abcam
SLC7A11	Rabbit	1:1000 WB	#12691	CST
ACSL4	Rabbit	1:1000 WB	ab155282	Abcam
GAPDH	Rabbit	1:5000 WB	ab181602	Abcam
Histone H3	Rabbit	1:1000 WB	#9715	CST
Goat anti‐rabbit IgG (HRP)	Goat	1:5000 WB	ab6721	Abcam
Goat anti‐rabbit IgG (H + L), Alexa Fluor 488	Goat	1:500 IF	A‐11008	Invitrogen

### 2.10 Immunofluorescence Staining

GES‐1 cells were seeded on sterile coverslips in six‐well plates and treated accordingly. Cells were washed with PBS and then fixed with 4% paraformaldehyde (Kermel, China) for 15 min, permeabilized with 0.3% Triton X‐100 (Absin, China) for 10 min, and blocked with 5% BSA (Kalang, China) for 1 h. After overnight incubation at 4°C with primary antibodies targeting Nrf2 or GPX4, cells were treated with fluorescently labeled secondary antibodies for 1 h in the dark. DAPI (Sangon, China) was used for nuclear staining, and fluorescence images were acquired using a microscope; the fluorescence intensity and localization were analyzed using ImageJ. Detailed information of antibodies was listed in Table 1.

### 2.11 Statistical Analysis

Experiments were independently repeated three times, and data are shown as mean ± SD. GraphPad Prism 8.0 was applied for statistical processing. Comparisons among multiple groups were made using one‐way ANOVA with LSD post hoc analysis. Significance was defined as *p* < 0.05.

## 3 Results

### 3.1 KAE Exhibits Favorable Safety and Attenuates H₂O₂‐Induced Cellular Injury

**Figure 1:**
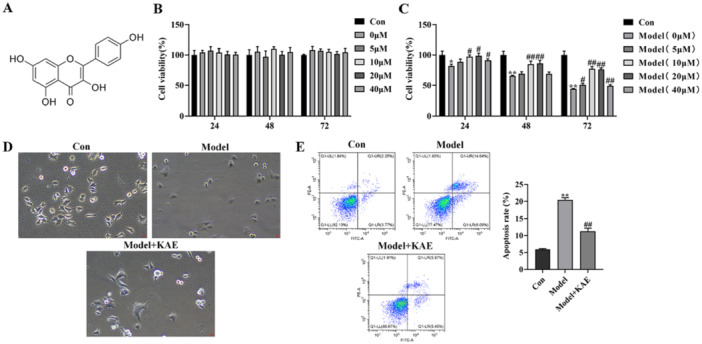
KAE exhibits no cytotoxicity and protects GES‐1 cells from H₂O₂‐induced injury. (A) Chemical structure of KAE. (B) CCK‐8 assay showing the toxicity of KAE concentrations (0–40 μM) on the viability of normal GES‐1 cells. (C) The protective effect of 0 μM, 5 μM, 10 μM, 20 μM, and 40 μM KAE on H₂O₂‐induced GES‐1 cells. (D) Representative light microscopy images showing morphological changes in GES‐1 cells under different treatments. (E) Evaluation of cell death by flow cytometry using Annexin V‐FITC/PI staining. Data are expressed as mean ± SD (*n* = 3). Scale bar = 20 μm. Data are presented as mean ± SD (*n* = 3 independent experiments). *p < 0.05, **p < 0.01 versus Con group; ^#^ *p* < 0.05, ^##^ *p* < 0.01 versus Model group.

The molecular structure of KAE is illustrated in Figure 1A. KAE has been previously reported to exert antioxidant and anti‐inflammatory effects. To investigate whether KAE confers similar protective effects in gastric mucosal injury, we first evaluated its potential cytotoxicity on GES‐1 cells using a CCK‐8 assay. Treatment with KAE in the range of 0−40 μM showed no notable impact on the viability of uninjured GES‐1 cells (Figure 1B), indicating that KAE is non‐toxic within this dose range. We then examined its protective effect against oxidative stress‐induced injury. CCK‐8 results demonstrated that KAE significantly improved cell viability following H₂O₂ exposure, with the most pronounced effects observed at 10 and 20 μM (24 h, *p* < 0.05; 48 and 72 h, *p *< 0.01; Figure 1C). Consequently, 10 μM was selected for subsequent experiments. Morphological observations under light microscopy revealed that KAE markedly alleviated H₂O₂‐induced cellular damage, including membrane shrinkage and cytoplasmic vacuolization (Figure 1D). Furthermore, flow cytometry analysis confirmed that KAE significantly reduced H₂O₂‐induced cell death (*p* < 0.01; Figure 1E). Collectively, these results indicate that KAE is well tolerated at commonly used concentrations and effectively mitigates oxidative stress‐induced injury in GES‐1 cells.

### 3.2 KAE Attenuates H₂O₂‐Induced Oxidative Stress and Ferroptosis

**Figure 2:**
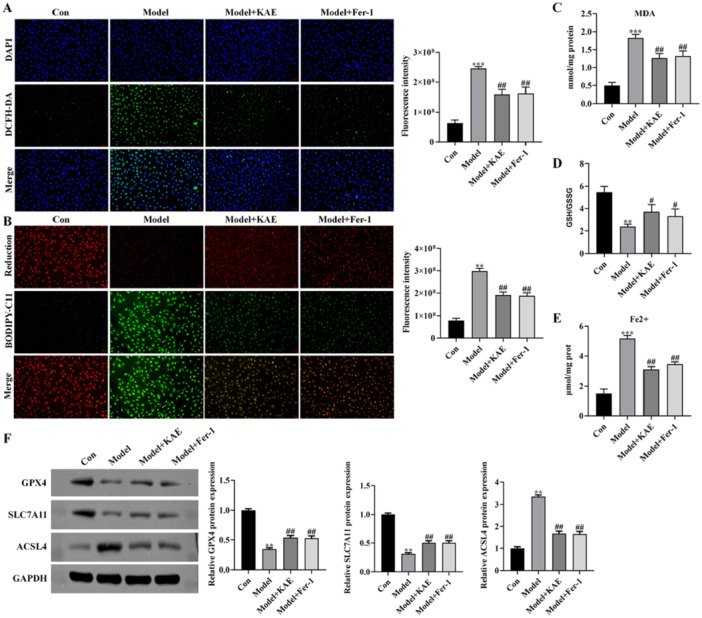
KAE alleviates oxidative stress and inhibits ferroptosis in H₂O₂‐treated GES‐1 cells. Con: control group; Model: H₂O₂‐treated group; Model + KAE: KAE‐treated group; Model + Fer‐1: ferrostatin‐1‐treated group. (A) Intracellular ROS levels measured by DCFH‐DA fluorescent probe. (B) Lipid peroxidation evaluated by BODIPY‐C11 staining. (C) MDA content determined by biochemical assay. (D) GSH/GSSG ratio reflecting antioxidant capacity. (E) Intracellular Fe²⁺ levels measured by colorimetric assay. (F) Western blot analysis of GPX4, SLC7A11, and ACSL4 protein levels. GAPDH was used as a loading control. Data are expressed as mean ± SD (*n* = 3). Scale bar = 20 μm. Data are presented as mean ± SD (*n* = 3 independent experiments). **p < 0.01, ***p < 0.001 versus Con group; ^#^ *p* < 0.05, ^##^ *p* < 0.01 versus Model group.

To gain deeper insight into how KAE protects GES‐1 cells from H₂O₂‐induced damage, four groups were established: control (Con), model (Model), KAE intervention (Model + KAE), and ferroptosis inhibitor treatment (Model + Fer‐1). Assessment of intracellular ROS was performed using the fluorescent probe DCFH‐DA. The results showed that KAE effectively suppressed H₂O₂‐induced ROS production, with a similar inhibitory effect observed in the Model + Fer‐1 group (*p *< 0.01; Figure 2A), suggesting that KAE may confer protection through the inhibition of ferroptosis under oxidative stress conditions. Lipid peroxidation was further evaluated using BODIPY‐C11 staining and MDA quantification. Both KAE and Fer‐1 dramatically reduced lipid peroxidation, as evidenced by decreased BODIPY‐C11 fluorescence intensity and MDA levels (*p *< 0.01; Figure 2B,C), indicating an attenuation of oxidative lipid damage. In addition, KAE markedly increased the intracellular GSH/GSSG ratio (*p *< 0.05; Figure 2D), reflecting enhanced cellular antioxidant capacity. Measurement of intracellular iron revealed that KAE significantly inhibited the excessive accumulation of Fe²⁺ induced by H₂O₂ exposure (*p *< 0.01; Figure 2E). Furthermore, Western blot analysis demonstrated that, similar to Fer‐1, KAE upregulated the expression of ferroptosis‐inhibitory proteins GPX4 and SLC7A11, while downregulating the pro‐ferroptotic protein ACSL4 (*p *< 0.01; Figure 2F). Collectively, these findings suggest that KAE protects gastric mucosal cells against H₂O₂‐induced injury by alleviating oxidative stress, reducing lipid peroxidation and iron accumulation, and ultimately inhibiting the ferroptosis pathway.

### 3.3 KAE Promotes Nrf2 Nuclear Translocation

**Figure 3:**
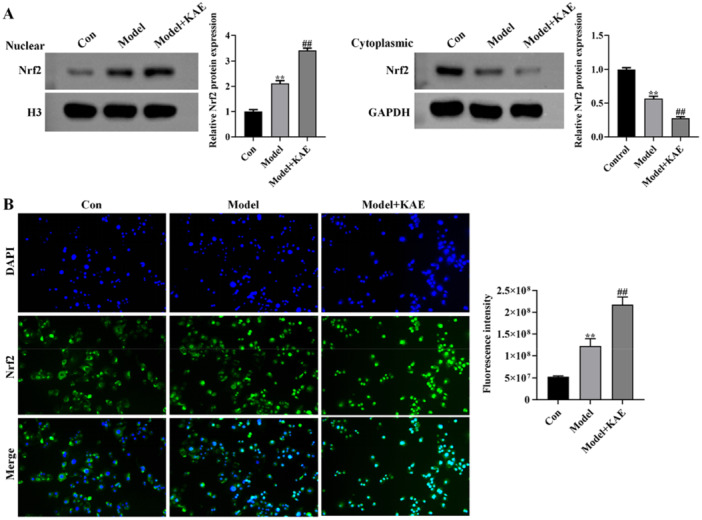
KAE promotes nuclear translocation of Nrf2 in GES‐1 cells. (A) Western blot analysis of cytoplasmic and nuclear Nrf2 levels following KAE treatment. GAPDH and Histone H3 were used as cytoplasmic and nuclear loading controls, respectively. (B) Immunofluorescence staining showing increased nuclear localization of Nrf2 after KAE treatment. Nuclei were counterstained with DAPI. Scale bar = 20 μm. Data are presented as mean ± SD (*n* = 3 independent experiments). **p < 0.01 versus Con group, ^##^ *p* < 0.01 versus Model group.

Oxidative stress triggers the release of Nrf2 from Keap1, allowing its nuclear translocation and subsequent initiation of antioxidant responses. To assess whether KAE confers its antioxidant effects by triggering the Nrf2 pathway, we examined the subcellular distribution of Nrf2 in GES‐1 cells. According to Western blot analysis, KAE significantly induced the cytoplasm‐to‐nucleus translocation of Nrf2, as indicated by increased nuclear Nrf2 expression and concomitant reduction in cytoplasmic Nrf2 levels (*p *< 0.01; Figure 3A), suggesting activation of the Nrf2 pathway. Immunofluorescence staining also confirmed that KAE enhanced the nuclear accumulation of Nrf2 (Figure 3B). These findings indicate that KAE can further facilitate the nuclear translocation of Nrf2 under oxidative stress, thereby potentially activating downstream antioxidant responses and providing a mechanistic basis for its protective effect against oxidative damage in gastric mucosal cells.

### 3.4 KAE Alleviates Oxidative Stress and Ferroptosis Triggered by H₂O₂ Through Nrf2/GPX4 Axis Activation

**Figure 4:**
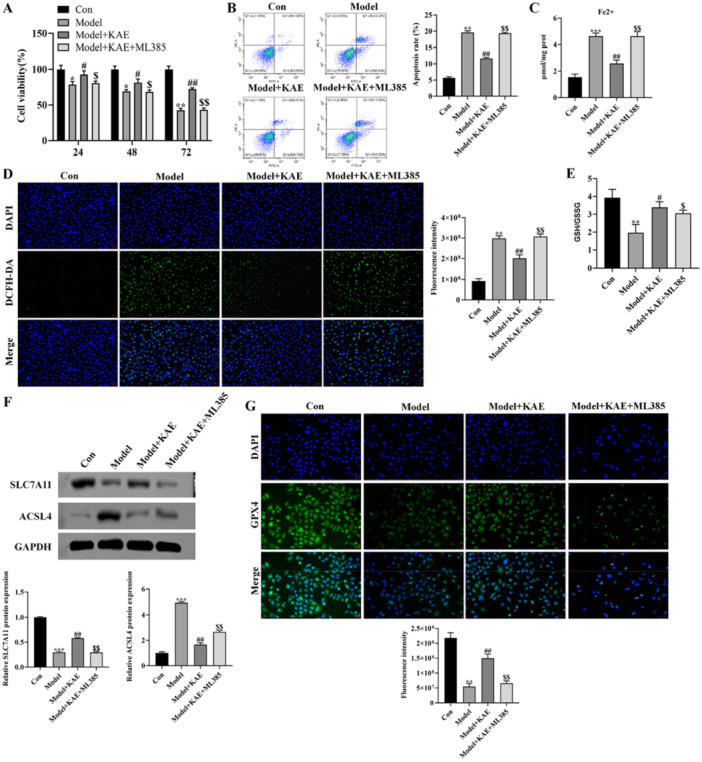
KAE attenuates oxidative stress and ferroptosis via activation of the Nrf2/GPX4 axis. Con: control group; Model: H₂O₂‐treated group; Model + KAE: KAE‐treated group; Model + KAE + ML385: Nrf2 inhibitor‐treated group. (A) CCK‐8 assay showing that the protective effect of KAE on cell viability is diminished by Nrf2 inhibitor ML385. (B) Flow cytometric analysis of cell death with and without ML385 co‐treatment. (C) Intracellular Fe²⁺ levels in different treatment groups. (D) Intracellular ROS levels and (E) GSH/GSSG ratio were measured to assess oxidative stress. (F) Western blot analysis of GPX4, SLC7A11, and ACSL4 expression under Nrf2 inhibition. (G) Immunofluorescence staining showing that ML385 suppresses KAE‐induced GPX4 expression. Data are expressed as mean ± SD (*n* = 3). Scale bar = 20 μm. Data are presented as mean ± SD (*n* = 3 independent experiments). *p < 0.05, **p < 0.01, ***p < 0.001 versus Con group; ^#^ *p* < 0.05, ^##^ *p* < 0.01 versus Model group; ^$^ *p* < 0.05, ^$$^ *p* < 0.01 versus Model + KAE group.

To further clarify whether the inhibitory effects of KAE on oxidative stress and ferroptosis are dependent on Nrf2/GPX4 axis, Nrf2‐specific inhibitor ML385 was employed for functional validation. The CCK‐8 results indicated that KAE treatment notably restored cell viability in H₂O₂‐exposed GES‐1 cells (24 and 48 h, *p* < 0.05; 72 h, *p* < 0.01), whereas this protective effect was markedly attenuated in the presence of ML385 (24 and 48 h, *p* < 0.05; 72 h, *p *< 0.01; Figure 4A). The reduction in cell death induced by KAE was markedly weakened upon ML385 treatment, as demonstrated by flow cytometric analysis (*p* < 0.01; Figure 4B). Moreover, the ability of KAE to suppress intracellular Fe²⁺ accumulation was significantly impaired by Nrf2 inhibition (*p* < 0.01; Figure 4C), indicating that Nrf2 activity is essential for KAE‐mediated regulation of iron homeostasis.

To further assess oxidative stress, we measured intracellular ROS levels and the GSH/GSSG ratio. KAE treatment significantly reduced ROS levels (*p* < 0.01) and increased the GSH/GSSG ratio (*p* < 0.05); however, these antioxidant effects were substantially weakened in the presence of ML385 (*p* < 0.01, *p *< 0.05; Figure 4D,E). Additionally, Western blot analysis showed that ML385 counteracted the KAE‐mediated upregulation of GPX4 and SLC7A11, as well as the downregulation of ACSL4 (*p *< 0.01; Figure 4F). Consistently, immunofluorescence staining further demonstrated that the KAE‐enhanced expression of GPX4 was significantly suppressed under Nrf2 inhibition (*p *< 0.01; Figure 4G). Collectively, these observations suggest that KAE protects gastric mucosal cells from H₂O₂‐induced injury via activating the Nrf2/GPX4 axis to suppress oxidative stress and ferroptosis.

## 4 Discussion

This study demonstrates that the natural flavonoid compound KAE inhibits ferroptosis by activating the Nrf2/GPX4 axis, thus shielding gastric mucosal cells from oxidative stress‐related injury. Beyond confirming the cytoprotective effect of KAE, these findings suggest that ferroptosis plays a mechanistically important role in oxidative stress‐induced gastric epithelial injury. These results provide novel perspectives for the development of natural therapies targeting gastric mucosal diseases, including gastric ulcers.

The pathogenesis of gastric mucosal damage is closely linked to oxidative stress. It not only disrupts cell membrane integrity but also causes an imbalance in intracellular iron homeostasis, facilitates ROS accumulation and lipid peroxidation, triggering ferroptosis and contributing to gastric mucosal cell damage and compromised tissue integrity [6]. KAE, a flavonoid compound present in many plants, has been shown to possess antioxidant and cytoprotective effects in various models of cellular injury. For instance, Xu et al. demonstrated that KAE alleviates oxidative damage in trophoblast HTR‐8/Svneo cells by modulating the NF‐κB signaling pathway [22]. Similarly, Yue et al. reported that KAE protects the heart by attenuating cobalt chloride‐induced myocardial cell injury via the HDAC3‐mediated Nrf2 pathway [23]. Here, we observed that KAE effectively promoted cell viability in gastric mucosal cells treated with H₂O₂, improved cellular morphology, and reduced cell death, without affecting the viability of normal GES‐1 cells. Our results support the notion that KAE protects gastric mucosal cells from damage. Given the central role of epithelial viability and redox balance in maintaining mucosal barrier integrity, our observations provide a plausible cellular basis for mucosal protection under oxidative stress.

A key finding of this study is that KAE alleviates oxidative stress‐induced gastric mucosal cell injury by targeting the ferroptosis pathway. Under oxidative stress conditions, elevated intracellular ROS levels, accumulation of lipid peroxides, increased Fe²⁺ load, and a decreased GSH/GSSG ratio are hallmark molecular features of ferroptosis [24]. Our results indicate that KAE treatment significantly mitigated these abnormalities, exhibiting effects comparable to the classical ferroptosis inhibitor, Fer‐1. Further mechanistic investigations revealed that KAE markedly upregulated the protein expression of GPX4, enhanced the expression of SLC7A11—a critical subunit of system Xc⁻, and downregulated the expression of ACSL4. As the only antioxidant enzyme known to directly neutralize lipid hydroperoxides within membranes, GPX4 is vital for preserving lipid balance and preventing ferroptosis [12]. SLC7A11 promotes cystine uptake, thereby facilitating GSH synthesis and providing the reducing substrate for GPX4, playing an upstream role in sustaining the cellular antioxidant environment [25]. In contrast, ACSL4 promotes the esterification of polyunsaturated fatty acids, enhancing ferroptosis susceptibility, and its downregulation helps mitigate lipid peroxidation [26]. Taken together, these findings indicate that KAE dampens an iron‐driven lipid peroxidation cascade under oxidative stress, thereby limiting ferroptosis‐associated epithelial injury.

At the signaling level, this study shows that KAE significantly promotes the nuclear translocation of the transcription factor Nrf2, thereby upregulating the expression of its downstream antioxidant target gene, GPX4. The nuclear localization of Nrf2, a critical mediator of antioxidant responses, is essential for triggering antioxidant gene expression [12]. To examine the contribution of Nrf2 to KAE's mode of action, we employed the specific Nrf2 inhibitor ML385 for co‐treatment. The findings revealed that blocking Nrf2 activity significantly weakened KAE's protective effects against oxidative stress and ferroptosis, reinforcing the pivotal role of the Nrf2 pathway in its cytoprotective function. Consistent with the work of Yuan et al. [25], our study supports the idea that KAE mitigates ferroptosis via Nrf2/SLC7A11/GPX4 activation, as previously demonstrated in neurons subjected to oxygen‐glucose deprivation/reperfusion. In addition, according to Ding et al., KAE increases the activity of antioxidant enzymes in H₂O₂‐treated SH‐SY5Y cells and alleviates oxidative stress via Nrf2‐dependent upregulation of heme oxygenase‐1 [27]. These results extend the role of Nrf2‐dependent ferroptosis regulation to gastric epithelial protection, providing a mechanistic link between antioxidant signaling and mucosal defense under oxidative stress.

Notably, the mechanism by which KAE inhibits ferroptosis through activation of the Nrf2/GPX4 axis is consistent with that reported for several other natural compounds. Previous studies have shown that natural products such as paeonol and eleutheroside can also effectively suppress ferroptosis through regulation of this signaling pathway [28, 29]. These results highlight the therapeutic potential of Nrf2 modulation in alleviating conditions associated with oxidative stress and ferroptosis. As a natural flavonoid compound with a stable structure, broad availability, and low toxicity, KAE exhibits promising pharmacological activity and safety, suggesting good prospects for clinical translation. Although this study focused on the Nrf2/GPX4 axis, other antioxidant and anti‐ferroptotic pathways may also contribute to the observed effects of KAE. For instance, inhibition of the NF‐κB pathway or modulation of MAPK signaling has been reported to enhance cellular resistance to oxidative stress in GES [5, 30]. Moreover, KAE has been shown in other models to influence Nrf2/HO‐1 signaling [15], suggesting a broader network of molecular interactions that warrants further investigation. In this regard, clarifying whether Nrf2/GPX4 activation cooperates with other stress‐response pathways (e.g., HO‐1 and NF‐κB/MAPK) will be important for defining the full protective mechanism of KAE and for informing subsequent in vivo validation.

In addition to our in vitro findings, recent in vivo studies have provided further evidence supporting the therapeutic potential of KAE in gastric diseases. For example, Gao et al. demonstrated that KAE inhibited the invasion and metastasis of gastric cancer cells in a mouse xenograft model by targeting the AKT/GSK3β pathway, thereby suppressing tumor growth and dissemination [31]. Although the pathological context differs from oxidative injury in gastric mucosal cells, these results highlight the broad spectrum of KAE's bioactivity in the stomach and suggest that it may confer protective or therapeutic benefits across diverse gastric disease models. Integrating such in vivo evidence with our current findings may help to bridge the gap between cellular mechanisms and potential clinical applications. The tumor model reflects biological processes that differ from those of acute oxidative epithelial injury, and thus, these in vivo data should be interpreted with caution when applied to gastric mucosal protection.

However, this study has certain limitations. First, it was conducted in an in vitro cell model, which cannot fully replicate the complexity of in vivo gastric mucosal injury. Specifically, the current GES‐1 model does not reflect key tissue‐level interactions present in gastric tissue, such as inflammatory cell infiltration, vascular responses, and systemic antioxidant regulation. Second, key pharmacokinetic properties—including its bioavailability, metabolic stability, and cellular uptake—were not assessed, and these will be important for translating in vitro efficacy into clinical relevance. Third, potential interactions of KAE with other antioxidant or anti‐ferroptotic pathways were not explored in depth. Lastly, standard mucosal protectants (e.g., Sucralfate, Rebamipide) were not included as positive controls, which limits direct efficacy comparisons and translational interpretation. Accordingly, future studies should incorporate pharmacokinetic evaluation, include standard mucosal protectants as comparators, and employ established animal models of gastric mucosal injury (e.g., ethanol‐, NSAID‐, or stress‐induced models) with assessment of ferroptosis‐related endpoints to confirm whether KAE confers mucosal protection in a physiologically integrated setting.

## 5 Conclusion

In summary, this study elucidates the protective mechanism by which KAE attenuates oxidative damage in gastric mucosal cells, highlighting its role in suppressing ferroptosis via Nrf2/GPX4 pathway activation. These results not only support the theoretical application of KAE as a gastric mucosal protectant but also provide novel perspectives and identify potential therapeutic targets for managing gastrointestinal diseases linked to oxidative stress. Nevertheless, further in vivo validation and comprehensive toxicity assessments are essential to confirm its safety and efficacy before clinical translation.

## Author Contributions

Zhiguang Sun and Chao Luo conceived the study and designed the experiments. Chao Luo completed the experiment, analyzed the data, and wrote the manuscript. Jing Yan, Yun Shen, and Xiong Xiao analyzed the data. Zhiguang Sun discussed the results and revised the manuscript.

## Ethics Statement

The authors have nothing to report.

## Consent

The authors have nothing to report.

## Conflicts of Interest

The authors declare no conflicts of interest.

## Data Availability

The data sets used and/or analyzed during the current study are available from the corresponding author on reasonable request.
